# A catch-up illusion arising from a distance-dependent perception bias in judging relative movement

**DOI:** 10.1038/s41598-017-17158-8

**Published:** 2017-12-06

**Authors:** Tobias Meilinger, Bärbel Garsoffky, Stephan Schwan

**Affiliations:** 1 Max Planck Institute for Biological Cybernetics, Max-Planck-Ring 8, 72076 Tübingen, Germany; 2 Leibniz-Institut für Wissensmedien, Schleichstraße 6, 72076 Tübingen, Germany

## Abstract

The perception of relative target movement from a dynamic observer is an unexamined psychological three body problem. To test the applicability of explanations for two moving bodies participants repeatedly judged the relative movements of two runners chasing each other in video clips displayed on a stationary screen. The chased person always ran at 3 m/s with an observer camera following or leading at 4.5, 3, 1.5 or 0 m/s. We harmonized the chaser speed in an adaptive staircase to determine the point of subjective equal movement speed between runners and observed (i) an underestimation of chaser speed if the runners moved towards the viewer, and (ii) an overestimation of chaser speed if the runners moved away from the viewer, leading to a catch-up illusion in case of equidistant runners. The bias was independent of the richness of available self-movement cues. Results are inconsistent with computing individual speeds, relying on constant visual angles, expansion rates, occlusions, or relative distances but are consistent with inducing the impression of relative movement through perceptually compressing and enlarging inter-runner distance. This mechanism should be considered when predicting human behavior in complex situations with multiple objects moving in depth such as driving or team sports.

## Introduction

How do moving observers perceive the relative movement between two objects? Despite its everyday relevance, this perceptual three-body problem is largely unexplored. For example, when driving on the highway, will a car on the lane to the right catch up with the car in front and therefore change into one’s own lane? Will the striker from one’s own soccer team catch-up with the opponent defender in time to reach a potential pass? Perceptual distortions may lead to a lost soccer game or a car accident.

Several mechanisms are involved in special cases of the current psychological three body movement problem. Visually moving observers parse out self-movement from the global optic flow pattern when estimating world-relative motion of a single object^1,2^. If available, also extra-retinal self-movement cues are used^3–5^. It is conceivable, yet not shown, that more self-movement cues will yield more accurate estimates also for estimating relative object motion.

Static observers judging the arrival time of multiple objects approaching a goal line overestimated the later-arriving object, but not the first-arriving one^6,7^. This indicates a bottleneck in processing object movement. Consequently, humans may not derive relative movement from spatial variables (i.e., perceived distances and velocities) of each object, but rely on visual field parameters, which has been demonstrated for various tasks such as breaking^8^, catching^9^, or self-movement perception^10^. This we have tested in the present study.

Relevant visual field parameters may focus on the direct relation between moving objects, for example, the visual angle or the distance between them, or may set them in relation to the observer, for example, depth distance of two moving objects relative to an observer. The latter can be visible in the frontal plane. For example, the border of occlusion on the farther object caused by the closer object is proportional to relative distance as are two projections into the frontal plane: the relative transversal distance of the objects to the line of sight and the relative vertical distance between the horizon and the point where an object touches the ground. Occlusion^11^ and visual distance to the horizon^12^ are important depth cues and keeping any of these relations constant is a potential strategy to judging objects as equally fast. To explicitly examine one of these parameters, we varied the presence of occlusion.

Visual angles (e.g., between objects or an object and the ground plane) are directly present within the visual field and are used for controlling actions such as catching a ball^9^. A straight forward strategy interprets changes in visual angle between objects as relative movement. For objects moving along a sphere of constant distance to the observer, this strategy will be largely correct. When moving in depth with equal speed, the visual angle between objects will change. Humans can compensate (sometimes even overcompensate) for the change in the visual angle with which an object is seen at different distances. For segments in the frontal plane seen under full cue conditions, this full compensation for observer distance can lead to perceptual size constancy^13^. However, for segments along the sagittal (viewing) axis, visual angle changes are typically not fully compensated; distances are compressed^14–21^, that is, perceived as shorter compared to vertically or transversally oriented segments at the same depth. This compression increases with observer distance. Gilinsky^18^ proposed a formula to express this increasingly larger compression: d = A*D/(A + D) where d is perceived distance, D the physical distance, and A the factor for how much perceived distance is governed by the visual angle (obtained for A → 0) or size constancy (A → ∞) and which varies with the viewing conditions (e.g., monocular vs. binocular). Several experiments support this proposed relation with observed A’s ranging from 7.6^14^ indicating large distance compression to 74^19^ indicating small distance compression and an medium A of about 28.5, originally proposed by Gilinsky^18^ (see also^16^). When two objects move away from an observer with equal speed, the inter-object distance will be increasingly more compressed and the impression of unequal movement speeds may occur. To compensate for compression, the closer object would have to move more slowly so that the physical inter-object distance increases in the same way as to cancel out increased compression. The size of A will determine the required speed reduction.

A last potentially relevant visual parameter is the visual expansion rate or the proportion of change in the visual angle taken up by an object. There is considerable evidence that humans and other animals rely on some version of this variable to judge time-to-contact and initiate appropriate actions^8,22,23^. Extending this parameter to the two-object case predicts that observers will perceive two objects with the same expansion rates as equally fast.

**Figure 1:**
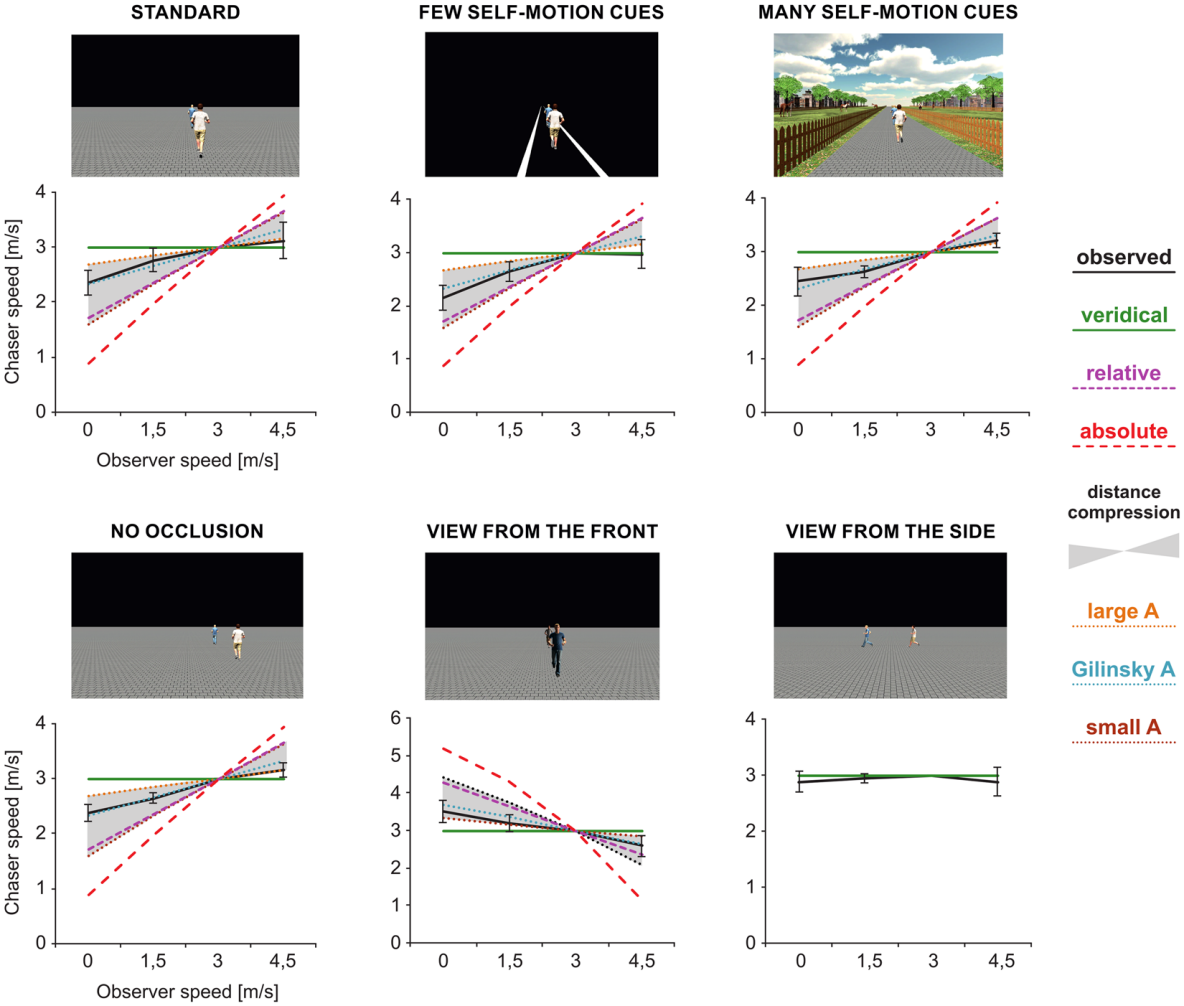
Experimental Stimuli and Results. Screenshots display the start configuration in the different experiments. Plots show the corresponding results and predictions. Results are comprised of mean observed values and 95% confidence intervals for points of subjective equality of chaser speed with the 3 m/s of the chased person as a function of observer speed. At an observer speed of 3 m/s, we normalized participants’ individual curves to 3 m/s to account for individual reaction biases. Shown predictions of observer speeds are due to small, average, and large distance compression, constant absolute visual angle between the runners, and constant relative distances which is almost identical to equal visual expansion rates for both runners and may be indicated by constant occlusions or constant relative distances of the runners to the line of sight or the horizon within the (frontal) screen plane.

**Figure 2:**
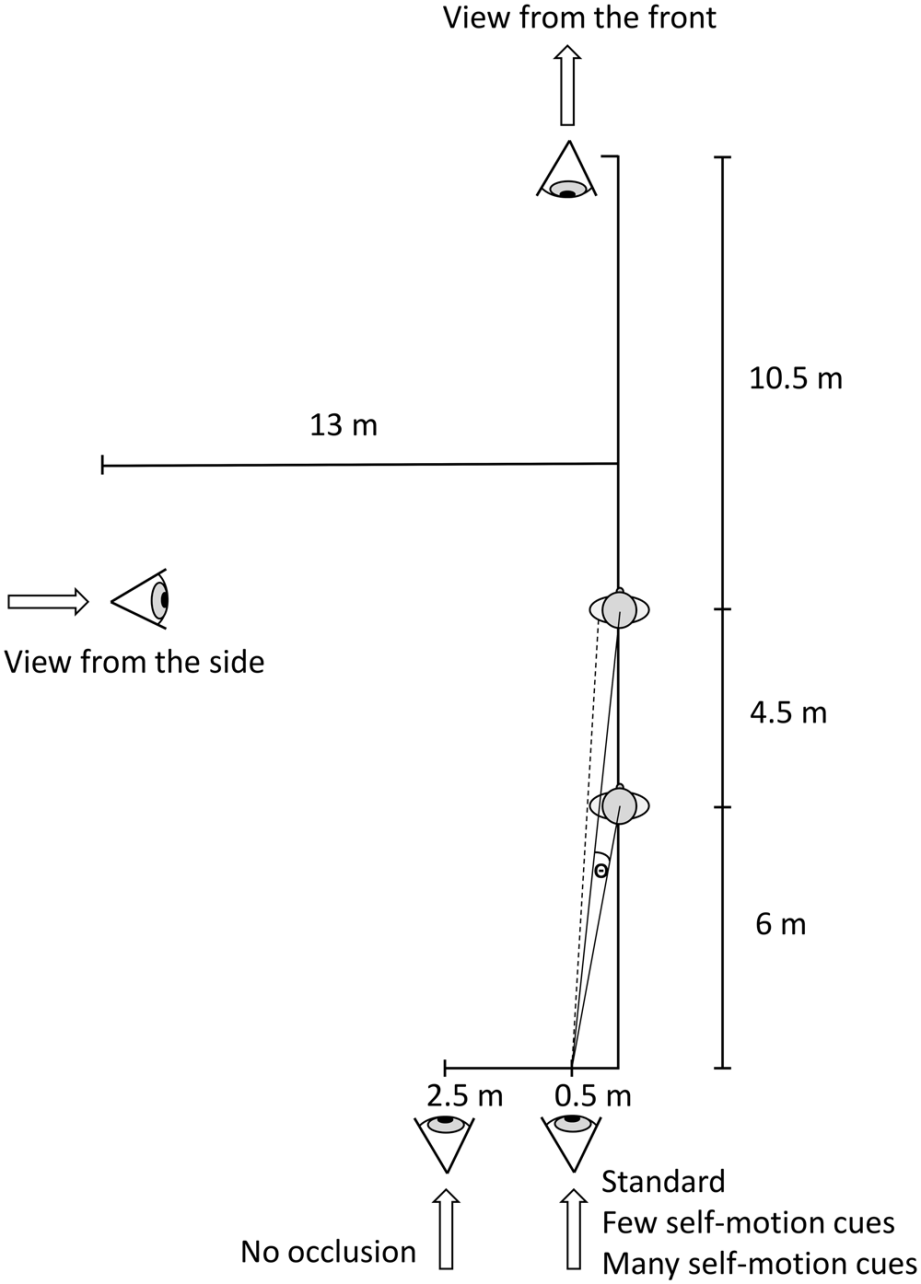
Drawing of the layout at start. The arrows indicate the direction of observer movement. Θ is the visual angle between runners. The dotted line indicates the occlusion area on the back of the avatar being chased.

We examined the influence of visual observer movement on the perception of relative movement. Participants watched 1.5 second video clips of a chaser running behind a chased person in the same direction (Fig. 1 and supplementary material for an illustration video). Participants watched the videos on a computer screen while sitting on a laboratory chair with their head on a chin rest matching the field of view of the video and the screen. The video scene with the runners was observed from behind, from the front, and for control, from the side by a static observer, or an observer visually approaching the scene (back and side view), or retreating from the scene (front view) with 50%, 100% or 150% of the speed of the chased person (i.e., 0, 1.5, 3, or 4.5 m/s) (Fig. 2). We varied chaser speed within adaptive staircases^24^ and participants indicated whether the chaser caught up or fell behind. We then adjusted the chaser speed to make both speeds perceptually more similar and repeated this procedure. From the fitted psychometric function, we determined the point of subjective equality where chaser and chased ran equally fast. We compared the observed velocities to predictions from keeping visual expansion rates, the absolute visual angle, or the relative distances between the runners constant and to predictions from small, medium, and large increased distance compression. We also varied the amount of self-movement information in order to examine whether self-motion would be parsed out. Finally, we varied occlusion between the runners to examine whether occlusion was used. Our results are best explained by small to medium distance compression.

## Results

Figure 1 shows the observed data under the different experimental conditions. When the avatars moved in depth (Exp. 1–5), participants did not perceive the speeds as veridical. The curves differ from predictions of veridicality in all experiments, five test with *F*(3,45) > 9.21, *p* < 0.001. When the runners were overall receding from the observer (areas below the line of veridicality e.g., low observer speeds with back camera and high speed with front camera), slower chaser speeds were perceived as being equally fast as the chased. Chaser speeds appeared to be quicker than expected by their physical speed, the illusion of catching up was induced. When the avatars were coming closer to the observer (above the line, slow speed with front camera, high speed with back camera), chaser speeds appeared to be slower, so that faster than physically correct chaser speeds appeared to be equally fast as the chased.

Predictions from constant relative distances, constant absolute visual angles, equal expansion rates, and large distance compressions significantly differ from the observed values in each experiment, 20 *F*(3,45) > 11.9, *p* < 0.001. Predictions from small distance compression do not fit the data for no occlusion, few and many self-movement cues, *F*(3,45) > 3.33, *p* < 0.028, other *F*(3,45) < 2.42, *p* > 0.079. Medium distance compression predictions deviate only for few self-movement cues, *F*(3,45) = 3.59, *p* = 0.021, other *F*(3,45) < 1.92, *p* > 0.139.

In all experiments, medium distance compression provides a better fit than all other models, 20 *F*(45,45) > 1.67, *p* < 0.045, except for small distance compression, five *F*(45,45) < 1.48, *p* > 0.096. Small distance compression provided a better fit than any model (other than medium compression) in all experiments, except for few self-movement cues, *F*(45,45) > 1.70, *p* < 0.040 in which it provided a better fit than keeping the visual angle constant, *F*(45,45) = 4.13, *p* < 0.001.

When the observer was looking from the side, all models predicted veridical performance and the data do not deviate from that prediction, *F*(3,45) = 1.10, *p* = 0.359.

In support for deviations from relative distance, we also did not observe differences in the shape of speed estimation curves values between of the standard experiment and the experiment with no occlusion, *F*(2,56) = 0.259, *p* = 0.773. Participants did not rely on occlusions for movement estimation.

We also varied the amount of self-movement cues in order to examine whether self-movement was estimated and parsed out. This variation did not change the shape of the speed estimation curves, *F*(4,84) = 0.479, *p* = 0.751, suggesting that self-movement speed was not a central part of the estimation process.

When looking at the speed estimates for observer speeds of 3 m/s, which was equal to the chased person’s speed and which we used to normalize individual curves, we observed an average underestimation of chaser speed between 0.058 m/s (from the side) and 0.47 m/s (standard), which was reliable for the standard experiment, *t*(14) = 2.83, *p* = 0.014, all other *t*(14) < 1.97, *p* > 0.069. This difference indicates an average response bias towards answering “fall behind”.

## Discussion

The perception of relative movement by a static or moving observer is a largely unexamined and yet common problem when driving or in multi-player ball games. With six experiments, we have shown that such movements are perceived veridically for movements in the frontal plane but biased for overall visual movements away from or towards an observer. With runners receding from an observer, slower chasers are perceived as being equally fast as the chased runner, suggesting that chasers appear to be quicker than expected by their physical speed. This yields the illusion of a chaser to catch-up with his physically equally fast chased runner. For runners overall approaching an observer the situation reverses. An equally fast catcher seems to fall behind. The size of the speed adjustment is inconsistent with all predictions except for medium^18^ and small^19^ distance compression - at least in some experiments. In all model comparisons, medium distance compression and in 75% of the comparisons also small distance compression fitted the data better than large distance compression^14^ and better than models based on visual field parameters. We tested models keeping the visual angle between the runners constant^9^, keeping the visual expansion rates of both runners or their in-between angle constant^8^, or keeping the relative distances or projections thereof into the frontal (screen) plane constant. These results were observed under a wide range of conditions: with and without occlusion, with varying self-movement cues, and when observing from the back or the front. We conclude that increased distance compression with observer distance biases the perception of relative movement which may influence choices in sports and traffic, for example, when overtaking a car on the slower lane which is perceptually not approaching the car in front but physically is and might thus move into one’s lane.

Our data are best accounted for by a small to medium increase in distance compression^18^. Will that be also the case for other situations, for example, when driving on the highway? The size of distance compression increase was shown to depend on the available situation, for example, the available depth cues^14–19,21^. With varying depth cues such as binocular instead of monocular vision^19^, different distance compressions and resulting perceptual biases might be observed. Also the presence of familiar size cues, which were shown to influence distance^13^, and speed perception^25^ may influence the bias although we did not find indication for that. Please note that the variations conducted to change self-movement information in the present experimentation mainly changed depth cues in the visual periphery. Depth cue changes targeting more directly at the runners themselves might also affect distance compression.

The lack of influence of self-movement cues in the present experimentation also indicates that world-relative self-movement estimation was not part of the judgments of relative movement. Prior studies have shown that self-movement was parsed out when judging the speed of a single object relative to the environment^1–5,26^. In the present case, observers relied on a strategy which did not take environment-relative movement into account and only relied on the inter-runner distance relative to the observer. The situation might clearly differ when the observer is moving physically not only visually and when no conflicting visual self-movement cues are present such as a static experimental room. Indeed, bodily motion cues were shown to combine with visual movement cues and even influence mere visual motion judgments for which they were completely irrelevant^27,28^. Future experiments will have to show whether physical self-motion will influence also relative motion judgments.

The present experimentation varied chaser speed and kept the speed of the chased runner relative to the environment constant. It is an interesting question for future experiments whether distance compression also predicts results from variations in the speed of the chased runner.

One main conclusion from the present work is that distance compression, a mechanism derived from the perception of static scenes, can be associated with relative motion in dynamic scenes. This makes the question for the underlying brain functions quite interesting. Will they encompass processes involved in static distance perception^29^, in target object or self-motion perception^30,31^, or both?

The observed bias in judging relative movement may have clear implications for driving and may potentially guide drivers to make dangerous driving decisions or guide players in ball games to play passes which will never reach their teammate. Potential countermeasures could involve explicit instruction to cognitively compensate for the perceptual bias, for example, while practicing for one’s driving license or for extensive training in team sports. Furthermore, car assistance systems and autonomous cars should be made aware of such a bias and the potentially resulting driving decisions to prevent dangerous and maybe even lethal situations.

## Methods

### Participants

Fifteen participants participated in each of the six experiments. Table 1 shows the distribution of their sex and age. We used mainly within-participant comparisons and an adaptive staircase method with varying step-sizes which provides precise measurements^24^. Here average effects should be detectable also with a moderate sample size. All participants were recruited via a subject database, were paid 8.- € for their participation, and signed an informed consent. The consent form and the experimental protocol were approved by the ethical committee of the Leibniz-Institute für Wissensmedien. All experiments were performed in accordance with relevant guidelines and regulations.

**Table 1:** Participant Numbers, Their Sex, and Average Age (*SD*) in the Different Experiments.

Experiment	Nr. of participants (female)	Age
1 Standard	15 (9)	25.67 (5.58)
2 Few self-motion cues	15 (10)	26.93 (11.23)
3 Many self-motion cues	15 (13)	25.47 (5.58)
4 No occlusion	15 (12)	22.47 (4.07)
5 View from the front	15 (12)	25.53 (9.36)
6 View from the side	15 (11)	24.93 (8.85)

### Setup and procedure

Participants watched 1.5 s long video sequences of two virtual humans (avatars) running in the same direction after each other (Fig. 1). Both Rocketbox© avatars’ animated running motion was identical, synchronized, and independent of the running speed. The speed of the chased person was fixed to 3 m/s and participants judged whether it escaped the catcher or not by pressing one of two keys on a keyboard. According to a staircase procedure described below, we adjusted the chaser’s speed to make both speeds perceptually more similar. The initial distance between both avatars was 4.5 m and the observer was 6 m away from the closest avatar when watching from the back, 10.5 m when watching from the front, and 13 m from the running trajectory when watching from the side (Fig. 2).

The observer was offset from the trajectory line by 0.5 m (standard) or by 2.5 m in the no occlusion experiment. The line of sight was parallel to the avatars’ orientations, their running trajectory, and the movement direction of the observer. When watching from the side, the observer’s line of sight at the start was directed at the avatar running away. We chose the initial observer distances in a way that for reasonable chaser speeds, arbitrarily defined here as 3 ± 2.5 m/s, both avatars were fully visible during the whole clip, but as close to the observer as possible. Both avatars were 1.72 m tall and the observer’s point of view was located at 1.65 m height. In pre-experiments, we did not observe any difference between observing from the left or the right of the runners, and therefore we only used the left side in walking direction. For the experiment with few self-motion cues, the ground plane was removed and only indicated by two sideline stripes (Fig. 1). The two runners were the only source of optic flow. The experiment with many self-motion cues provided a lot of contrast for optical flow, motion parallax, and familiar size cues.

The setup was programmed in Unity 4.6.1 and ran on an Apple Mac mini A1347. Participants watched the videos on a Pro Lite B2776HDS-W2 27″ TFT screen with a resolution of 1600 × 900 pixel. While watching within a half-darkened laboratory room, they rested their head on a chinrest 62 cm away from the screen. The resulting visual field of view of the scene of 51° × 30° was identical to the field of view of the virtual observer in the virtual scene.

During the 1.5 s clip, the observer moved at a speed of 0, 1.5, 3, or 4.5 m/s. Participants judged whether the chaser would catch up with the escaper or fall behind. The speed was adjusted according to the procedure laid out below until reaching criterion. Participants practiced the procedure on two test trials. A longer example video with equidistant runners showing the catch-up illusion is found in the supplementary materials.

### Staircase procedure and analysis

For measuring the perceived equal chaser speed to the movement of the runner being chased as well as the just noticeable differences (JND), we used a 1-up-1-down, a 2-up-1-down, and a 2-down-1-up adaptive staircase procedure for each observer speed^24^. For example, for the 2-down-1-up staircase, we started with an obviously faster chaser speed of 5.5 m/s. When participants judged the chaser as faster twice in a row, we decreased the chaser speed by 0.2 m/s. However, when the chaser was judged as being slower, we immediately increased the speed by the same amount. After eight reversals in speed adjustment (e.g., faster last time, but slower now, or vice versa), we halved the step size of speed adjustment to 0.1 m/s in order to measure the point more precisely. After eight additional reversals with the smaller step size, the staircase procedure ended for this staircase. This algorithm converges to the point where the probability of judging “faster” twice equals the probability of judging “slower” at least once within two trials. The probability for a single faster choice is therefore given by the square root of 0.5 which is 0.707. For this stimulus, a participant will judge in 70.7% of the cases the chaser as faster and in 29.3% of the cases as slower. The 2-up-1-down staircase worked in reverse direction: starting with chaser speed of 0.5 m/s, increasing speed after two successive slower judgments, but decreasing it after one faster judgment again for two times eight reversals targeting at the point with 29.3% faster and 70.7% slower judgments. The 1-up-1-down staircase started with 0.5 m/s and increased or decreased the speed immediately after each judgment. It had the same eight reversals with 0.2 m/s and eight reversals with 0.1 m/s as the other reversals and targeted directly at the point of subjective equality (PSE) where 50% faster/slower judgments are given.

We repeated the three staircases for each observer speed, resulting in overall twelve staircases per participant. These were presented intermingled in random order. In each block, all staircases not yet completed were presented in newly chosen random order. The overall procedure lasted until all twelve staircases reached the stop criterion of two times 8 reversals. On average, this procedure resulted in 651 (SD = 64) trials per participant.

**Figure 3:**
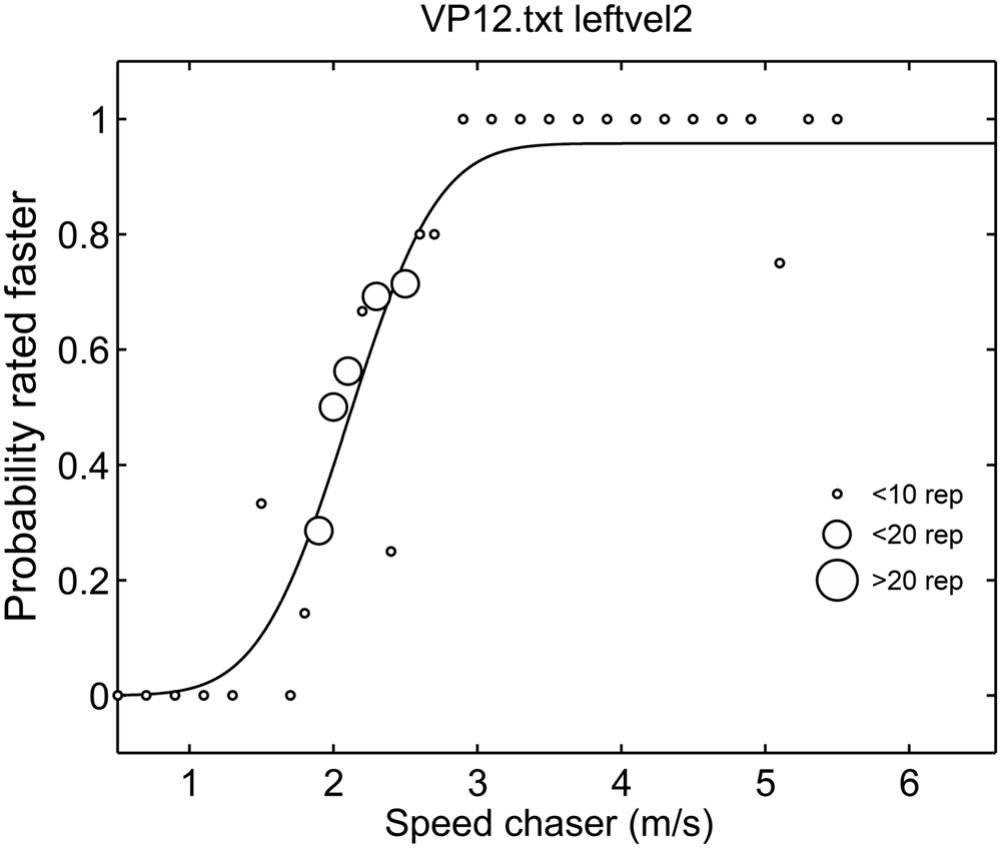
Exemplary data from a participant with the fitted psychometric function.

All judgments for a single observer speed (i.e., data from all three staircases) were pooled and we fitted a psychometric function (Fig. 3). This curve consisted of a cumulative Gaussian with two additional lapse rates to account for stimulus unrelated errors^32^. For example, the psychometric curve in Fig. 3 reaches an asymptotic value of judging the chaser as being faster which is slightly lower than 100%. We fitted the curve in Matlab using a least square error procedure, with the lapse rates constrained to values between 0 and 0.05. We used the 50% point of the curve as an estimate for the PSE and its standard deviation as an estimate for the JND.

The described procedure with three staircases targeted at chaser speeds resulting in 29.3%, 50%, and 70.7% faster judgments is suitable not only for determining the PSE but also the JND which expresses the dispersion of subjective equality or how sharply a participant distinguishes the speed differences as shown in the slope of the curve at the PSE. We estimated PSEs and JNDs for each participant in each observer speed condition. We did not observe differences in JNDs except for the observer at the side where values increased with observer speed in accordance with previous work^33^. JNDs are not reported further; we used only individual PSEs for further analysis.

### Computation of the prediction curves

The following formulas can be used to compute the different predictions.

Notations (compare Fig. 2):

*D*_1_: Distance observer - chaser at start

*D*_2_: Distance observer – chased person at start

${D}_{1}^{^{\prime} }$: Distance observer - chaser at end

${D}_{2}^{^{\prime} }$: Distance observer – chased person at end

*B* =$\,B^{\prime}$ distance observer - line of avatar movement

‘ denotes the visual geometry at the end

When the observer is in front, chaser and chased are reversed as is observer movement.

#### Distance compression

Perceptual distance from physical: (1) $d=\frac{A\ast D}{(A+D)}$ (Gilinksy, 1951) whereas $A,\,D\in {\mathbb{R}}$

Physical distance from perceptual: (2) $D=\frac{A\ast d}{(A-d)}$

Assumption: (3) $${d}_{2}-{d}_{1}=\,{d}_{2}^{^{\prime} }-{d}_{1}^{^{\prime} }$$

(4) $${d}_{1}^{^{\prime} }={d}_{2}^{^{\prime} }-({d}_{2}-{d}_{1})$$

(5) $${D}_{1}^{^{\prime} }=\frac{A\ast {{\rm{d}}}_{1}^{^{\prime} }}{(A-{{\rm{d}}}_{1}^{^{\prime} })}$$

We used the lowest A = 7.57^14^, the highest A = 74^19^, and as an average, the original A = 28.5 obtained in multiple experiments^16,18^ for prediction.

#### Absolute/constant visual angle

Assumption: (6) $${\Theta }={\Theta }^{\prime} $$

Start: (7) $${\Theta }={{\Phi }}_{2}-{{\Phi }}_{1}=a\,tan\frac{{D}_{2}}{B}-a\,tan\frac{{D}_{1}}{B}$$

End: (8) $${\Theta }^{\prime} ={{\Phi }}_{2}^{^{\prime} }-{{\Phi }}_{1}^{^{\prime} }=a\,tan\frac{{D}_{2}^{^{\prime} }}{B}-a\,tan\frac{{D}_{1}^{^{\prime} }}{B}$$

(9) $$\iff \,a\,tan\frac{{D}_{1}^{^{\prime} }}{B}=a\,tan\frac{{D}_{2}^{^{\prime} }}{B}-{\Theta }$$

(10) $$\Rightarrow \,{D}_{1}^{^{\prime} }=\,\tan (a\,\tan \,\frac{{D}_{2}^{^{\prime} }}{B}-{\Theta })\ast B$$

Please note that the predicted values slightly change according to whether horizontal theta (where the base is B as indicated in Fig. 2) or vertical theta is used (where the base B is the observer height of 1.65 m). Throughout the paper, we have used the horizontal case as laid out in Fig. 2. This allows for capturing prediction changes in the experiment without occlusion with the larger base B of 2.5 m. Using vertical theta instead did not change any of the result patterns but just changed some numerical values.

#### Relative/Relative distance

Assumption: (11) $$\frac{{D}_{2}}{{D}_{1}}=\frac{{D}_{2}^{^{\prime} }}{{D}_{1}^{^{\prime} }}$$

(12) $$\Rightarrow {D}_{1}^{^{\prime} }=\frac{{D}_{1}\ast {D}_{2}^{^{\prime} }}{{D}_{2}}$$

According to the intercept theorem, the same relation applies to ratios of vertical and transversal distances of projections of the corresponding points of the two objects into the frontal plane such as the distance of the participants’ feet from the horizon, the line of sight (vertical middle line in pictures of Fig. 1), or the proportion of the back of the avatar running away which is not occluded by the chaser.

#### Visual expansion rate (inverse tau)

Avatar height: (13) *H* = 1.72 *m*

Visual angle: (14) ${{\Theta }}_{H}={\rm{a}}\,\tan \,\frac{H}{D}$ Comment: approximation as the line of sight is not at the bottom or top of the avatar.

Change in visual angle: (15) ${\Delta }{{\Theta }}_{H}={{\Theta }}_{H}-{{\Theta }}_{H}^{^{\prime} }$

Proportion change in visual angle: (16) $\frac{{{\rm{\Delta }}{\rm{\Theta }}}_{H}}{{{\Theta }}_{H}}$

Assumption: (17) $$\frac{{\Delta }{{\Theta }}_{H1}}{{{\Theta }}_{H1}}=\frac{{\Delta }{{\Theta }}_{H1}}{{{\Theta }}_{H1}}$$

(18) $$\iff \frac{{{\rm{\Theta }}}_{H1}-{{\rm{\Theta }}}_{H1}^{^{\prime} }}{{{\rm{\Theta }}}_{H1}}=\frac{{\Delta }{{\Theta }}_{H2}}{{{\Theta }}_{H2}}$$

(19) $$\iff \frac{a\,tan\frac{H}{{D}_{1}}-\,a\,tan\frac{H}{{D}_{1}^{^{\prime} }}}{a\,tan\frac{H}{{D}_{1}}}=\frac{{\Delta }{{\Theta }}_{H2}}{{{\Theta }}_{H2}}$$

(20) $$\iff \,a\,tan\frac{H}{{D}_{1}^{^{\prime} }}=a\,tan\frac{H}{{D}_{1}}-\frac{{\Delta }{{\Theta }}_{H2}}{{{\Theta }}_{H2}}\ast a\,tan\frac{H}{{D}_{1}}$$

(21) $$\Rightarrow \frac{H}{{D}_{1}^{^{\prime} }}=\,\tan (a\,tan\frac{H}{{D}_{1}}\,-\,\frac{{\Delta }{{\Theta }}_{H2}}{{{\Theta }}_{H2}}\ast a\,tan\frac{H}{{D}_{1}})$$

(22) $$\iff {D}_{1}^{^{\prime} }=\frac{H}{\tan (a\,tan\frac{H}{{D}_{1}}-\,\frac{{\Delta }{{\Theta }}_{H2}}{{{\Theta }}_{H2}}\ast a\,tan\frac{H}{{D}_{1}})}$$

Please note that tau uses the inverse of proportion change in visual angle ${{\rm{\Theta }}}_{H}/{{\rm{\Delta }}{\rm{\Theta }}}_{H}$, but as this ratio has to be kept constant, the predictions do not change. The alternative of keeping the expansion rate of the visual angle between the runners constant does not seem possible. For constant chaser speeds smaller than 5 m/s, expansion rate seems to change considerably within the 1.5 s interval and thus cannot be kept constant.

#### Getting the predicted speed from D1′

Observer speed: (23) *v*_*obs*_ = [0, 1.5, 3, 4.5]

Video clip duration: (24) ${\Delta }t\,=1.5\,s$

Position change in visual field: (24) ${D}_{1}^{^{\prime} }-\,{D}_{1}$

World-relative position change: (25) ${D}_{1}^{^{\prime} }-{D}_{1}+{v}_{obs}\ast {\rm{\Delta }}t$

Predicted speed for chaser: (26) ${v}_{chaser}=\frac{{D}_{1}^{^{\prime} }-{D}_{1}+{v}_{obs}\ast {\rm{\Delta }}t}{{\rm{\Delta }}t}$

The numerical predictions are summarized in Table 2.

**Table 2:** Numerical Predictions for Chaser Speed According to the Different Theories in *m*/*s* in the Standard Experiment.

*Theory*	*Observer speed [m/s]*			
	0	*1.5*	3	*4.5*
Veridical	3	3	3	3
Distance compression				
Small: A = 74	2.68	2.84	3	3.15
Medium: A = 28.5	2.32	2.67	3	3.32
Large: A = 7.57	1.59	2.32	3	3.63
Equal expansion rates	1.77	2.38	3	3.61
Relative distance const.	1.71	2.36	3	3.64
Absolute visual angle const.	0.83	1.95	3	3.96

### Statistical Analysis

We normalized individual PSE curves at observer speed of 3 m/s to account for individual reaction biases and erased values deviating more than 3 standard deviations from the overall mean in an experiment (one value for observer in front). For comparing the data with the veridical speeds and the model predictions, we used lack of fit tests. Lack of fit compares deviation between observed values and local average per speed condition with the deviation of the local average and the predicted value with an F-test. To *compare model fits*, we used F-tests evaluating the ratio of mean squared errors between two model predictions for each experiment. Finally, we examined the interaction between experiment and speed condition in mixed measures ANOVAs to compare the observed curves between experiments. Raw data (Supplementary Dataset 1) as well as PSEs (Supplementary Dataset 2) are found in the supplementary materials.

### Data availability

The authors declare that the data supporting the findings of this study are available within the paper and its supplementary information files.

## Electronic supplementary material

Supplementary Dataset 2

Supplementary Dataset 1

Supplementary Illustration Video

Supplementary Information 
