# Supplementary material for: A catch-up illusion arising from a distance-dependent perception bias in judging relative movement
Source: Sci Rep. 2017 Dec 6;7:17037. doi: 10.1038/s41598-017-17158-8 (PMC5719034; doi:10.1038/s41598-017-17158-8)
Supplement: Supplementary file 4 — Supplementary Information [file 41598_2017_17158_MOESM4_ESM.pdf]

A catch-up illusion arising from a distance-dependent perception bias in judging relative movement

Tobias Meilinger, Bärbel Garsoffky, and Stephan Schwan

Supplementary information

Supplementary Illustration Video. Two runners move away from a static observer while keeping the same distance between each other. However, the chaser seems to catch up illustrating the catch-up illusion. The videos in the experiment ended after 1.5 seconds and chaser as well as observer speed varied.

Supplementary Dataset 1. Raw data. “VELOCITY_1-4“ correspond to camera speeds of 0, 1.5, 3 and 4.5 m/s respectively. “alternations“ indicate direction changes in a staircase with the current step size. FALSE in “2up1down“ identifies a 1up1down staircase. Here, TRUE can also mean 2down1up depending on the direction specified in “stcsType“. Speed is given in units of 0.1 m/s (e.g., 30 is 3.0 m/s). One sheet contains the data of one participant. The first number in the sheet name indicates the experiment number as in Figure 1 or Table 1.

Supplementary Dataset 2. PSEs and predictions. The comma-separated values file contains variables and values. Experiment order is as in Figure 1 or Table 1.
